# Composition of Three Common Chinese Herbal Medicines and the Influence of Preparation Types on the Bioaccessibility of Trace Elements

**DOI:** 10.3390/toxics10120719

**Published:** 2022-11-24

**Authors:** Xiaoming Wan, Weibin Zeng

**Affiliations:** 1 Institute of Geographic Sciences and Natural Resources Research, Chinese Academy of Sciences, Beijing 100101, China; 2 University of Chinese Academy of Sciences, Beijing 100049, China

**Keywords:** arsenic, *Astragalus*, decoct, distribution, *Glycyrrhiza*, *Isatidis*, micro-XRF, sulfur

## Abstract

The high concentration of trace elements in Chinese herbal medicine (CHM) is an important research topic for quality control. This study investigated the total concentration of trace elements in three herbs used as both medicine and supplementary food, including *Astragalus membranaceus*, *Glycyrrhiza*, and *Isatidis.* Further, the effects of different preparation ways, such as decoct, granule, and oral liquid, on the bioaccessibility of trace elements in CHM were disclosed. Results indicated that the total concentrations of trace elements in these three herbs were lower than the medical standards, but the concentrations of As and Pb in CHMs were higher than the standards for supplementary food. Different preparations ways affect bioaccessibility. Powder and oral liquid show a high bioaccessibility possibly because of the grinding process and the repeated extraction with ethanol. Among the three different CHMs, Isatidis showed higher bioaccessibility of As, which may be related to the sulfur fumigation process of this CHM. The three investigated CHMs were found to be safe as medicine but presented risks as supplementary food. The apparent influence of preparation procedures on the bioaccessibility of trace elements indicated that it is necessary to appropriately regulate preparation processes for CHMs.

## 1. Introduction

Chinese herbal medicine (CHM) is popular across the globe as dietary supplements, and traditional and alternative medicine [1,2]. CHM has a clinical effect on vascular dementia [3], cancer immune regulation [4], diabetic nephropathy [5], and hepatocellular carcinoma [6].

Recently, the inclusion of CHM in the Chinese protocol for combating the coronavirus disease (COVID-19) has been effective because of its efficacy and comprehensive therapeutic theory [7,8]. CHM can reduce the incidence of severe or critical events, improve the clinical recovery of patients with COVID-19, and helps alleviate symptoms such as cough or fever, likely through its host-directed regulation and certain antiviral effects [9]. CHMs have advantages such as abundant clinical experiences, and their unique diversity of chemical structures and biological activities.

Health risks from CHMs present serious concerns. Poisoning by metals and metalloids at concentrations above acceptable regulatory standards has been reported [10]. CHM showed different degrees of trace element contamination, which is affected by different types of CHM, medicament portions, elements, preparations, and CHM sources [11]. In a survey on 247 CHMs, 5–15% of the samples contained higher-than-standard concentrations of arsenic (As), and ~5% of them contained higher-than-standard concentrations of lead (Pb) [12].

Earlier studies on the potential risks associated with CHMs mostly focused on the total concentration of trace elements, in which a limited number of studies focus on the bioaccessibility of trace elements in CHMs, which could reflect the actual levels of trace elements exposed to the human body [13]. A better understanding of the bioaccessibility of trace elements is crucial for the assessment of their health risk against humans.

Currently, herbal medicines are frequently used in various dosage forms, while consumers do not focus on the particulars of the traditional dosage forms [14]. Herbs are dispensed in the form of decoct, powder, granule, or oral liquid. During CHM production, the use of traditional or modern processing methods results in a risk of trace element contamination throughout the whole procedure [15]. Whether these different processing methods affect the bioaccessibility of trace elements in CHMs is unclear.

*Astragalus membranaceus*, *Glycyrrhiza uralensis*, and *Isatidis radix* are three medicine food homologous plants that are the most frequently applied herbs in the world. The root of *A. membranaceus* is a type of CHM widely used as a health-promoting agent with a long history in China, and it has multiple functions, including the regulation of immune function, antioxidant, anti-aging, antitumor, anti-fibrosis, antibacterial, reducing blood glucose, lowering blood lipid, neuroprotectivity, and hepatoprotectivity [16,17]. The dried roots and rhizome of *G. uralensis* are often used for the treatment of diseases such as weakness of the spleen and stomach, fatigue, palpitation, shortness of breath, cough and phlegm, epigastrium, limb contracture, acute pain, and carbuncle; it is commonly used to alleviate drug toxicity [18]. *I. radix* is an important and commonly used drug for clearing heat and detoxification, cooling blood, and regulating the pharynx, and it has been used in China and Asia for thousands of years; it has played an important role in preventing and alleviating the symptoms of SARS (Severe Acute Respiratory Syndrome) [19]. *G. uralensis* and *I. radix* are also essential compositions for the Lianhuaqingwen capsule. Lianhuaqingwen capsules can improve the improvement rate of clinical symptoms of COVID-19, such as fever, fatigue, cough, improve lung imaging lesions, shorten the duration of symptoms, and improve the clinical cure rate [20].

To confirm whether different preparation types of CHMs could affect the bioaccessibility of trace elements in CHMs, we prepared three of the most commonly used herbal CHMs, namely, *A. membranaceus*, *G. uralensis*, and *I. radix*, in different ways, and analyzed for the bioaccessibility of trace elements. Results could provide information on better regulation of the safe utilization of CHMs.

## 2. Materials and Methods

### 2.1. CHM Materials and Analysis of Total Concentration of Trace elements

**Figure 1:**
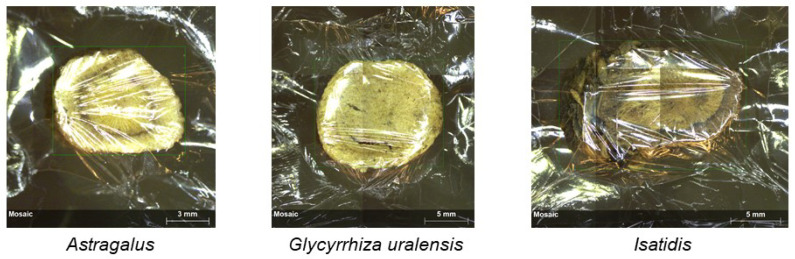
Experimental herbal medicines.

Dry roots of *A. membranaceus, G. uralensis,* and *I. radix* originating from Gansu Province, China was selected as the CHM materials (Figure 1). They were identified according to the Pharmacopoeia of China, and the voucher specimens were preserved in a specimen cabinet at the Institute of Geographical Sciences and Natural Resources Research, Chinese Academy of Sciences (Beijing, China).

The total concentrations of trace elements in CHMs were analyzed by grinding the plant roots and digesting them with a mixture of HNO_3_–HClO_4_ (4:1, *v*/*v*) [21,22]. There were six replicates for each CHM material.

### 2.2. Micro-XRF Analysis for the Distribution of Elements and the Inorganic Compositions

The high-resolution distribution of different mineral elements was obtained using a high-performance micro-X-ray fluorescence spectrometer (Bruker, M4 TORNADO PLUS, Berlin, Germany) [23,24,25]. Roots were fixed to the sample stage by using an X-ray sample film (Prolene* Thin-film, gauge: 0.00016,” 4 µm; 40,640). The analysis parameters were set in accordance with the manufacturer’s instructions as follows: X-ray beam spot size, ≤20 μm for Mo–K; step size, 15 μm; scanning time for each step, 20 ms; excitation, high-brilliance X-ray tube with polycapillary X-ray optics, target material, Rh; 50 kV, 600 μA; vacuum path; and silicon drift detector, detector energy resolution <145 eV. A map scan was made to measure the relative content of inorganic elements, including Al, O, Mg, Na, Si, P, S, K, Ca, Mn, Fe, Cu, Zn, and Cl, of the three CHMs.

### 2.3. Preparation Types of CHMs

Three different preparation types were designed for the CHMs.

(1) Decoct: the raw CHM material was placed in a bottle with 678.8 mL of ultrapure water, soaked for 40 min, and then boiled for 3 min once every 40 min. This process was repeated thrice. The volume of the solution was controlled within 200 mL and centrifuged at 5000× *g* for 5 min.
(2) Powder: the raw CHM material was first ground to powder (<150 μm), and the same procedure for decoction was followed.
(3) Granule: the raw CHM material was soaked and boiled twice using the same procedure as a decoction, and then 95% ethanol was added to the decoction until the ethanol content reached 70%. The supernatant was collected and condensed into a 10 mL extract. The extract was mixed with dextrin, sucrose, and 95% ethanol, dried at 50 °C for 15 min, and granulated into particles with the size of <1.18 mm.
(4) Liquid: the raw CHM was soaked and boiled twice by using the same procedure as decoction and condensed to an extract with a density of 1.08–1.12 g/cm^3^. The supernatant was collected, added with 95% ethanol until the ethanol content reached 60%, and condensed again to an extract with a density of 1.30–1.33 g/cm^3^. The supernatant was mixed with sucrose syrup (60%) and diluted to a volume of 1000 mL.

To analyze the total concentrations of trace elements in different preparation types, the solution obtained above was digested with a mixture of HNO_3_–HClO_4_ (4:1, *v*/*v*) [21,22].

### 2.4. Extraction and Analysis for the Bioaccessible Fraction of Trace Elements in CHMs

Bioaccessible fraction extraction included two steps [13]. During the first step, 0.5 g material was mixed with 30 mL of simulated gastric fluid, shaken and extracted in an incubator-rotary at 30 rpm for 1 h at 37 °C, and then centrifuged at 3500× *g* for 5 min. The supernatant was collected and condensed to approximately 3 mL at a low temperature by using an electro-thermal plate.

Then, the residue from step 1 was extracted using simulated intestinal fluid via the same procedure. The supernatant was collected and condensed using the same procedure as step 1. Two parts of the supernatant were measured for the concentration of trace elements.

The simulated gastric fluid was 1.25 g pepsin, 0.50 g sodium citrate, 0.50 g sodium malate, 500 μL of acetic acid, and 420 μL of lactic acid made up to 1 L by deionized water. The pH was adjusted to 2.0 by 30% HCl.

The simulated intestinal fluid consisted of 1.75 g bile salts and 0.5 g pancreatin made up to 1 L by deionized water. The pH was adjusted to 7.0 by NaHCO_3_.

To analyze the total concentrations of trace elements in different extracts, the solution obtained above was digested with a mixture of HNO_3_–HClO_4_.

### 2.5. Chemical Analysis and Quality Control

The As concentrations were measured via atomic fluorescence spectrometry (Haiguang AFS-2202, Beijing Kechuang Haiguang Instrumental Co., Ltd., Beijing, China). The concentrations of cadmium (Cd), chromium (Cr), nickel (Ni), Pb, and zinc (Zn) were measured via inductively coupled plasma–mass spectrometry (ICP–MS; ELAN DRCe; PerkinElmer, Shelton, CT, USA). For quality control, the samples of certified standard reference materials for plants (GBW07603) from the China National Standard Materials Center were digested with the experimental samples. The recovery rates of trace elements were 95~110%.

## 3. Results

### 3.1. Total Concentration of Trace Elements in CHMs

**Figure 2:**
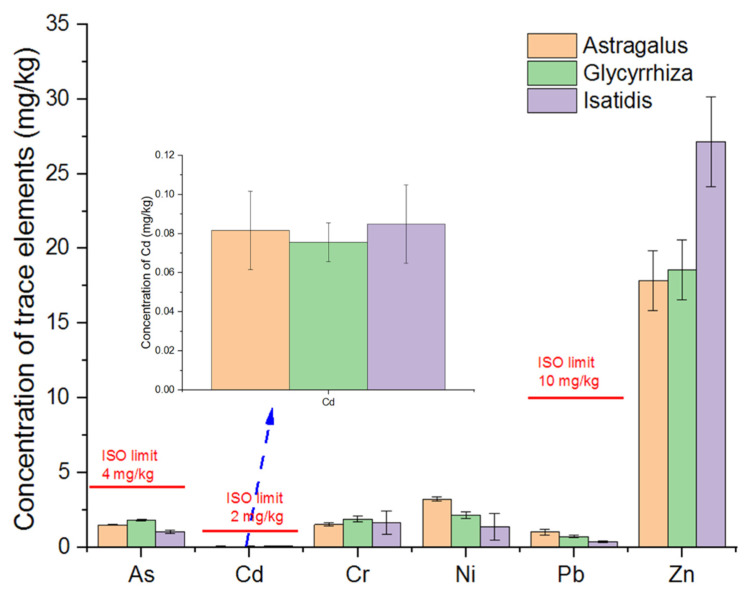
Total concentrations of trace elements in CHMs (mg/kg).

According to Figure 2, the total concentrations of As, Cd, and Pb in CHMs do not exceed the ISO standard [26]. The total concentrations of trace elements in CHMs in the current study were lower than all the standards listed in Table 1.

The concentration of different trace elements varied. The concentration of Zn was the highest, while the concentration of Cd was the lowest. In terms of different herbs, *I. radix* had the highest Cd and Zn concentration, but low As, Ni, and Pb concentration. *G. uralensis* had the highest Cr concentration, while *Astragalus* had the highest Ni and Pb concentration.

Notably, the three CHMs, namely, *A. membranaceus*, *G. uralensis*, and *I. radix,* were both medicinal and edible plants. Although the total concentrations of trace elements in CHMs were lower than the medicine standards (ISO 18664 2015), the concentrations of As and Pb in all the three CHMs were higher than the maximal pollutant concentration regulated for food in the National Food safety standards—quantity of pollutants in food (GB 2762-2017) [27]. The concentration of As in all three CHMs was higher than the food standard limit of 0.5 mg/kg for supplementary food regulated by GB 2762-2017. The concentration of Pb was also higher than that regulated by GB 2762-2017 (0.5 mg/kg for formula food with special medical use).

### 3.2. Bioaccessible Fractions of Trace Elements after Different Preparation Ways

**Figure 3:**
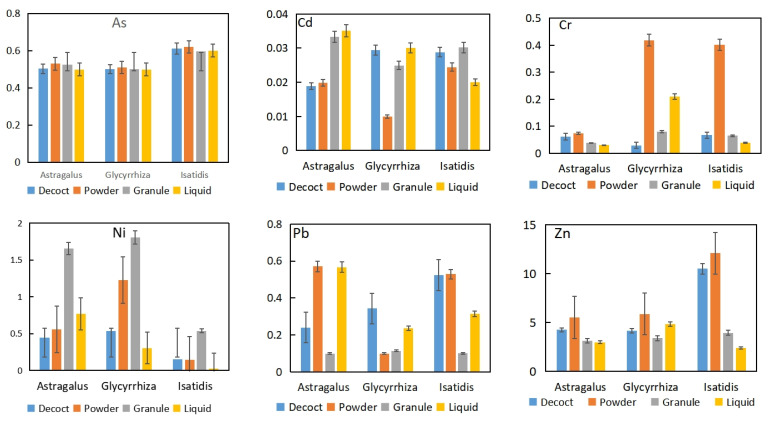
Concentrations of the bioaccessible trace elements in CHMs (mg/kg).

Bioaccessible fractions of trace elements varied in different herbs and among different preparation types (Figure 3).

The difference in the bioaccessible concentration of As did not have a significant difference among the four preparation types (*p* > 0.05) but showed a difference among the three herbs, and the As concentration in *Isatidis* is slightly higher than that of the two other herbs. For Cd, trends were different in these three herbs. Granule and liquid indicated higher inaccessibility of Cd than decoct and powder in *Astragalus*. Powder led to a higher bioaccessibility of Cr and Zn in all three herbs, while granule led to a higher bioaccessibility of Ni. Powder showed the highest bioaccessibility for Cd and Zn, while granule showed the highest bioaccessibility of Ni, and oral liquid showed a lower bioaccessibility of trace elements, except Cd. Among the three herbs, *Isidia* indicated a slightly higher concentration of bioaccessible As and Zn than the other two herbs.

**Figure 4:**
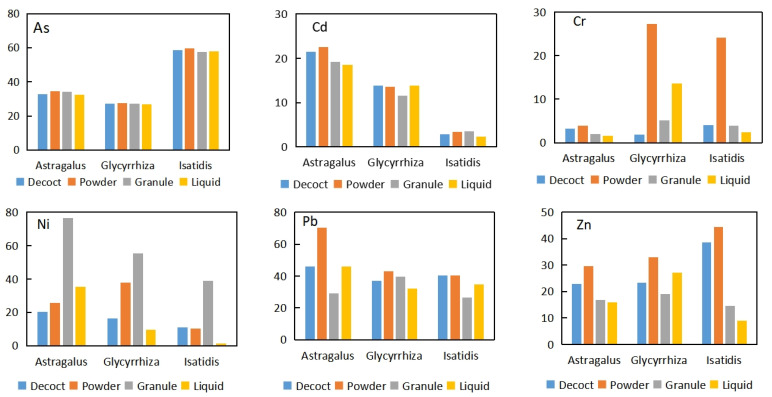
Percentage of the bioaccessible fractions to the total concentration of trace elements in CHMs (%).

The percentages of the bioaccessible fractions of Ni and Pb were higher than other trace elements, with the highest percentage being ~80% (Figure 4), while the bioaccessible fraction of Cr was the lowest, which is less than 10%. The bioaccessible fraction of As, Cd, and Zn was medium, with percentages of 25–45%, 3–20%, and 20–74%, respectively. For As and Cd, no apparent difference was observed in the bioaccessible fraction percentage among different preparation types. For Cr, Pb, and Zn, powder resulted in the highest bioaccessible fraction. For Ni, granule led to the highest bioaccessible fraction. In terms of the difference among herbs, the percentage of the bioaccessible fractions of As was significantly higher in *Isatidis* than the two other herbs, while *Astragalus* had a high Cd bioaccessibility.

### 3.3. Inorganic Compositions of CHMs and Their Spatial Distribution

The main inorganic compositions of the three herbs are described in Table 2. Three of the top elements were O, Si, and K for *Astragali,* and O, Ca, and K for *Glycyrrhizar* and *Isatis*, respectively (Table 2). Fe, Zn, Cu, and Mn are four of the most abundant trace elements in these three herbs (Table 2). The difference in the chemical composition of these three herbs is not apparent. *Astragali* had a significantly higher concentration of Fe but a lower concentration of Ca, and *G. uralensis* had a significantly higher concentration of Ca and Mg but a lower concentration of P and Fe. *Isatis* had significantly higher concentrations of S and Zn but lower concentrations of Si.

Correlation analysis indicated significant positive correlations among several elements (Table 3). Therefore, the inorganic elements considered in the current study had a similar trend.

**Figure 5:**
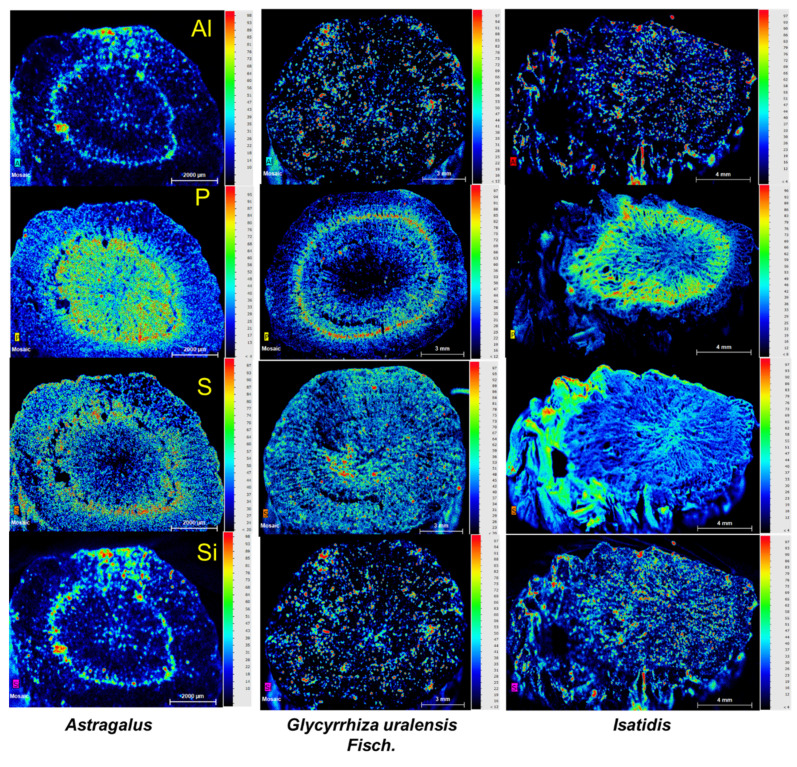
Distribution of Al, P, S, and Si on the three experimental herbal medicines, imaged using a μ-XRF spectrometer. Normalized X-ray fluorescence intensities are scaled from red (maximum) to blue (minimum) as indicated at the right.

Al, P, S, and Si in the Kasparian strip were enriched for *Astragalus*, as shown in Figure 5, but this trend was only found for P in the two other herbs. Notably, S was enriched in the surface of *Isatidis* and is higher than the two other herbs.

**Figure 6:**
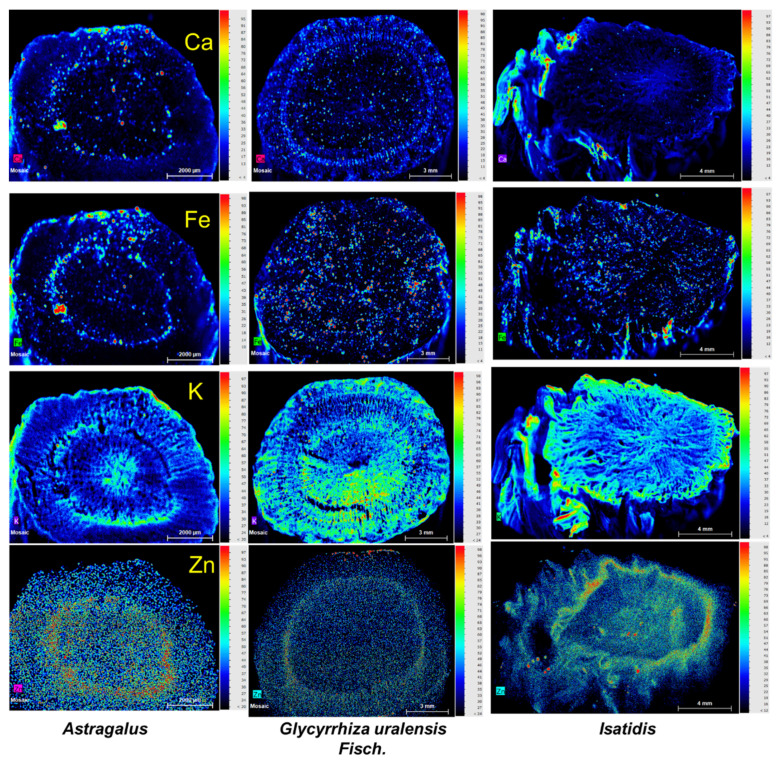
Distribution of Ca, Fe, K, and Zn on the three experimental herbal medicines, imaged using a μ-XRF spectrometer. Normalized X-ray fluorescence intensities are scaled from red (maximum) to blue (minimum) as indicated at the right.

Similarly, in *Astragalus*, the enrichment of Ca, Fe, K, and Zn in the Casparian strip was apparent. For *Glycyrrhiza*, Ca and Zn are enriched in the Casparian strip (Figure 6). For *Isatidis*, only Zn showed this pattern.

Similar to S, Zn was higher in *Isatis* than the two other herbs (Table 2). However, the spatial distribution of Zn was different from S (Figure 6). Zn was enriched in the Casparian strip for all these three herbs.

## 4. Discussion

### 4.1. Potential Health Risks of Trace Elements in CHMs as Dietary Supplements

The results of the current study were within the same range as the published studies. The total concentrations of Pb and Cd recorded in *Astragali* were 8 and 0.85 mg/kg, respectively [28]. The contents of Cd, Pb, and Zn in *Astragali* were 0.08, 0.63, and 4.14 mg/kg, respectively [29]. The concentration ranges were 0.03–0.32 mg/kg for As, 0.003–0.072 mg/kg for Cd, and 0.05–3.52 mg/kg for Pb in *Glycyrrhiza Uralensis* [30]. In terms of Isatidis, the Zn concentration is 29.7 mg/kg, Ni concentration is 1.53 mg/kg, whereas, Cr was not detected [31].

The results in the current study were in accordance with a large-scale survey on trace element concentrations, indicating that the levels of trace elements in the vast majority of the Chinese-grown herb samples were likely to be of negligible concern [32].

Although these three CHMs are safe for medical use, the concentration of As and Pb in all three CHMs was higher than the food standard limit of 0.5 mg As/kg for supplementary food and 0.5 mg Pb/kg for formula food with special medical use. *A. membranaceus*, *G. uralensis*, and *I. radix* were all included in the list of “Both Food and Medicine Substance” issued by National Health and Family Planning Commission of the People’s Republic of China. Only the names of herbs are included in the list, without other definitions or descriptions. Standards and rules should be formulated for the safe use of this list, considering both the health and safety principles [33]. *A. membranaceus* is commonly used as an ingredient for stewed soup. Through the long-time boiling process, the extra trace elements in this herb may cause harm to human beings. Further, the extracts of these three herbs were commonly sold as health products. The long-term consumption of these herbs may also cause potential harm to human beings.

### 4.2. The Comparatively Higher Bioaccessibility of as in Isatidis May Result from Sulfur Fumigation

The content of S was high in *Isatidis* (Table 2), and S showed an enrichment trend on the surface of *Isatidis* (Figure 5). S on the surface of *Isatidis* roots may be enriched by sulfur fumigation. Considering the pesticidal and anti-bacterial properties of sulfur fumigation, it was used to replace traditional sun-drying cheaply and conveniently [34]. Although this method has been prohibited in China since 2004, cases of S fumigation of herb medicines are still recorded.

Further, with S fumigation, the volatile toxic elements such as Hg or As might migrate to the surface of the medicine, and thus change the existing form of toxic elements in herbs [35]. According to bioaccessibility analysis, the bioaccessible fraction of As was significantly higher in *Isatidis* than in the two other herbs (Figure 3 and Figure 4). Results of S and As indicated that S fumigation may occur in *Isatidis*, which improved the bioaccessibility of As.

However, considering the high detection limit of the desktop μ-XRF, the distribution of trace elements was not obtained in the current study. Further high-desolation synchrotron-radiation-XRF analysis of As may provide more information.

Notably, although *Isatidis* also contained a high concentration of Zn (Table 2), the spatial distribution of Zn was different from that of S, implying that the higher concentration of Zn in *Isatidis* was not caused by the processing but from the herb itself.

### 4.3. Preparation Ways for the CHMs

The results of the current study indicated that different preparation ways led to varied trace element bioaccessibility caused by the varied extraction solvent and processes used.

The preparation methods for CHMs included in this study are common methods used in the CHMs formulations. Decoct or prolonged boiling is the earliest and most popular method for the preparation of herbal medicines in the practice of CHM. Grinding is included in powder preparation to increase the medicine’s efficacy. Granule and oral liquid may further add other subsidiary materials such as sucrose.

Results indicated that powder had a higher bioaccessibility for most treatments, thus supporting that the grinding procedure increased its contact with the extraction solution. Only Ni indicated a high bioaccessibility under granule and oral liquid preparations (Figure 3), which might be related to the addition of other materials during the preparation process.

Although decoct is the most popular preparation method, decoct may be gradually replaced by other preparations such as granule, because of quality control of the herbal ingredients, the time and inconvenience they are required to prepare, and their transportation and storage are hard to ensure. Granule preparation is currently recommended for clinical use because they are safe and certainly simple to control, produce and manage as a consistent medical product than decoctions [36]. Considering the increased Ni bioaccessibility, standardization of granules and rigorous pharmacological, toxicological, and clinical studies are needed to demonstrate its equivalence or advantage compared with decoctions.

### 4.4. Appropriate Evaluation Criteria of Trace Elements in CHMs

The total concentrations of trace elements in the CHMs are lower than the related standards (Figure 2). The bioaccessible fraction accounted for less than 80% of the total concentration. No standard regulates the bioaccessible fraction of trace elements in CHMs.

The inherent characteristics of traditional Chinese medicine are multi-components and multi-targets, which lays a theoretical foundation for the multi-purpose use of traditional Chinese Medicine [37]. The toxicity of traditional Chinese medicine was also regarded by some experts as an important part of traditional Chinese medicine [38]. In addition, sometimes there are positive interactions between organic components and trace elements [39]. However, despite the possible beneficial effects of trace elements in CHMs, the quality control of CHM is important in CHM application. Moreover, the appropriate regulation of trace elements, especially toxic ones, is important.

This report was the first to indicate that the different preparation processes of CHMs could lead to apparent differences in the bioaccessibility of trace elements. The biggest difference was 10 folds. The different preparation processes also led to differences in the efficacy of CHMs. Different drying and extraction methods result in different losses of essential compounds from the herbs [40]. Therefore, the preparation process and its related effect on bioaccessibility should be involved in the future regulation of the trace element contents in CHMs.

## 5. Conclusions

The three CHMs investigated in this study indicated the acceptable total concentration of trace elements for medical use, but the concentrations of As and Pb exceeded the National food safety standards. Different preparations ways affect the bioaccessibilities of CHMs. Powder and oral liquid show high bioaccessibility, possibly because of the grinding process and the repeated extraction using ethanol. Notably, sulfur fumigation may exist for Isatidis, which improved the bioaccessibility of As for this CHM. The results could be useful for the further regulation of trace elements in CHMs.

## Figures and Tables

**Table 1:** The standard for Chinese medicinal materials in terms of the total concentrations of trace elements (unit: mg/kg).

Element	Pb	As	Cd	Cu
China	5	2	1	
WHO	10		0.3	
Import and export *	5	2	0.3	20
ISO18664 2015	10	4	2	
Taiwan authority	5	3	1	
Hongkong	5	2	1	20
Japan	20	5		10
USA	5		0.5	
India	10	3	0.3	
Canada	10	5	0.3	2

* Indicates the Green Trade Standards of Importing & Exporting Medicinal plants & Preparations, China.

**Table 2:** Inorganic compositions of different CHMs (%).

	*Astragalus*	*Glycyrrhizae*	*Isatis*
Al	2.47 ± 0.12	1.94 ± 0.08	1.15 ± 0.08
O	73.7 ± 1.3	70.3 ± 5.8	72.2 ± 5.9
Mg	0.67 ± 0.02	1.15 ± 0.10	0.55 ± 0.06
Na	0.22 ± 0.05	0.48 ± 0.07	0.15 ± 0.04
Si	8.14 ± 058	7.18 ± 1.01	3.99 ± 0.19
P	1.03 ± 0.06	0.65 ± 0.08	1.26 ± 0.22
S	0.48 ± 0.04	1.10 ± 0.04	3.23 ± 0.26
K	9.46 ± 1.24	7.03 ± 1.01	9.90 ± 1.05
Ca	2.42 ± 0.11	8.82 ± 1.25	6.29 ± 0.99
Mn	0.04 ± 0.01	0.04 ± 0.01	0.05 ± 0.01
Fe	1.08 ± 0.05	0.75 ± 0.08	0.82 ± 0.10
Cu	0.01 ± 0.01	0.01 ± 0.01	0.01 ± 0.01
Zn	0.03 ± 0.01	0.03 ± 0.01	0.08 ± 0.01
Cl	0.28 ± 0.02	0.55 ± 0.09	0.34 ± 0.06

**Table 3:** Correlation among inorganic elements in CHMs.

	Al	O	Mg	Na	Si	P	S	K	Ca	Mn	Fe	Cu	Zn
O	0.893 *												
Mg	0.841 *	0.852 *											
Na	0.765	0.730	0.980 **										
Si	0.996 **	0.901 *	0.884 *	0.817 *									
P	0.718	0.920 **	0.584	0.410	0.705								
S	0.185	0.606	0.334	0.192	0.203	0.764							
K	0.832 *	0.976 **	0.716	0.562	0.826 *	0.981 **	0.677						
Ca	0.551	0.761	0.884 *	0.855 *	0.612	0.578	0.639	0.649					
Mn	0.837 *	0.991 **	0.785	0.645	0.841 *	0.961 **	0.692	0.992 **	0.737				
Fe	0.937 **	0.970 **	0.763	0.630	0.926 **	0.915 *	0.478	0.970 **	0.585	0.959 **			
Cu	0.838 *	0.833 *	0.999 **	0.986 **	0.882 *	0.553	0.296	0.691	0.872 *	0.761	0.746		
Zn	0.415	0.765	0.424	0.254	0.416	0.918 **	0.955 **	0.847 *	0.610	0.842 *	0.693	0.387	
Cl	0.763	0.868 *	0.979 **	0.942 **	0.811	0.645	0.506	0.749	0.957 **	0.822 *	0.745	0.971 **	0.559

* Indicates a significant correlation at the level of 0.05. ** indicates a significant correlation at the level of 0.01.

## Data Availability

The datasets generated during and/or analyzed during the current study are not publicly available but are available from the corresponding author on request.
